# The Effect of Proton Pump Inhibitors on Bone Mineral Density at Specific Anatomical Sites: A Systematic Review and Meta‐Analysis

**DOI:** 10.1155/bmri/1269905

**Published:** 2025-12-29

**Authors:** Areeg Anwer Ali, Bhoomendra A. Bhongade, Fatima Mohamed Alkaabi

**Affiliations:** ^1^ Department of Clinical Pharmacy and Pharmacology, RAK College of Pharmacy, Ras Al Khaimah Medical & Health Sciences University, Ras Al Khaimah, UAE; ^2^ Department of Pharmaceutical Chemistry, RAK College of Pharmacy, Ras Al Khaimah Medical & Health Sciences University, Ras Al Khaimah, UAE

**Keywords:** bone mineral density, meta-analysis, proton pump inhibitor, systematic review

## Abstract

There have been some concerns about the potential adverse consequences of proton pump inhibitors (PPIs) on bone health, specifically with respect to bone mineral density (BMD). The objective of the present investigation was to evaluate the impact of PPIs on BMD in the lumbar spine, femoral neck, and total hip regions. On the basis of PRISMA, a systematic review and meta‐analysis were performed. PubMed, Scopus, Cochrane Library, and Google Scholar were used to find eligible observational studies published between January 2010 and January 2025. The ROBINS‐I tool was employed to evaluate potential for bias, and studies with critical bias were excluded. To synthesize the data, random‐effects models were utilized, and the *I* ^2^ statistic was used to evaluate heterogeneity. The sensitivity analyses and publication bias were also performed. A systematic review and quantitative synthesis used 20 and seven records, respectively, of 170 records screened. The comprehensive pooled analysis revealed that the decrease in BMD with the use of PPIs was modest yet statistically significant (SMD −0.15, 95% CI −0.21 to 0.09) alongside a substantial degree of heterogeneity (*I* ^2^ = 93.6*%*). Subgroup analysis demonstrated that there were significant decreases at the femoral neck (SMD −0.27, 95% CI −0.46 to −0.09), but not at the lumbar spine or the total hip. The funnel plot analysis indicated a certain level of asymmetry, and the sensitivity analysis indicated that the results were mostly robust; unless the study that excluded one outlier was used in the analysis, then the lumbar spine results would change. The use of PPIs is related to a significant but relatively small decrease in BMD, most obvious at the femoral neck, although findings across anatomical locations are heterogeneous. The results of this study support the cautious use of PPIs in all people at risk of osteoporosis and the need to conduct a high‐quality prospective study to understand site‐specific effects in the future.

## 1. Introduction

Proton pump inhibitors (PPIs) are among the most frequently prescribed medications in clinical practice due to their potent and long‐lasting suppression of gastric acid secretion. They are widely used in the management of gastroesophageal reflux disease (GERD) and for the prevention of NSAID‐induced gastrointestinal injury, largely because of their favorable safety profile and rapid symptom relief [1–3].

Several PPIs, including omeprazole, esomeprazole, lansoprazole, rabeprazole, and pantoprazole, are FDA‐approved and share the same fundamental mechanism of irreversibly inhibiting the gastric H+/K+‐ATPase pump (proton pump), although they differ in pharmacodynamic and pharmacokinetic properties such as bioavailability, metabolism, and duration of action, contributing to interindividual differences in therapeutic response. These inactive prodrugs are weak bases with a pKa value around 4 and are slightly protonated at neutral pH. They become activated only in the highly acidic environment of the parietal cell canaliculi, where higher protonation occurs and are converted into their active sulfenamide form [4, 5].

Despite the clinical benefits of PPIs, they can cause abdominal pain, headaches, constipation, diarrhea, and flatulence. Moreover, the long‐term PPI therapy has been associated with a variety of adverse outcomes, such as the increased likelihood of community‐acquired pneumonia, stomach carcinoids, gastric neoplasia, kidney disease, dementia, fractures, *Clostridium difficile* infection, hypomagnesemia, drug–drug interaction, and depression [6–11]. These concerns are amplified by the frequent and sometimes prolonged use of PPIs without well‐defined guidance on treatment duration or monitoring. Differences in acid suppression among individuals—driven partly by genetic polymorphisms affecting CYP450 metabolism—further complicate optimal dosing strategies [12].

One area of growing scientific and clinical concern is the potential impact of long‐term PPI use on skeletal health. Several epidemiological studies have reported associations between PPI exposure and increased fracture risk, particularly involving the hip, spine, and wrist [13]. While observational data do not definitively establish causation, the consistency of associations across multiple populations has prompted investigation into the biological pathways through which PPIs might affect bone health. Several mechanisms have been proposed. One hypothesis suggests that gastric acid suppression reduces intestinal calcium solubility and absorption, leading to negative calcium balance over time. Another posits that long‐term PPI use results in hypergastrinemia, which may stimulate parathyroid activity or influence bone turnover. Additionally, PPIs may exert direct effects on osteoclast proton pumps, potentially altering bone resorption processes [14]. However, the extent to which these mechanisms contribute to clinically meaningful and uniform reductions in bone mineral density (BMD) across different skeletal sites, such as the lumbar spine (LS), femoral neck (FN), or total hip (TH), remains unclear.

Several systematic reviews and meta‐analyses have examined this association with mixed conclusions [14–16]. Some studies suggest modest reductions in BMD among PPI users, while others report clinically negligible changes [17–19]. This inconsistency may be explained by differences in study design, duration of follow‐up, population characteristics, and methods of BMD assessment. Furthermore, some reviews have combined results from various anatomical sites or relied on heterogeneous outcome measures, making it difficult to determine whether PPIs exert site‐specific effects on skeletal density [18–20].

A recent umbrella review synthesizing data from 27 systematic reviews highlighted that PPI use has been linked to alterations in BMD across different skeletal regions but emphasized that the clinical significance of these findings remains uncertain, particularly in relation to fracture risk reduction or acceleration [17]. Importantly, many previous reviews have focused primarily on fractures as an endpoint rather than on changes in BMD itself. While fracture risk and BMD are related, they are not interchangeable; fractures result from a complex interplay of bone strength, fall risk, comorbidities, and medication exposures. Therefore, isolating BMD as a specific outcome is essential to understanding the direct skeletal effects of PPIs.

To date, no comprehensive systematic review or meta‐analysis has exclusively examined the effects of PPI use on BMD at the three most clinically relevant anatomical sites: LS, FN, and TH. These sites are of particular importance because they are the primary regions assessed in dual‐energy X‐ray absorptiometry (DXA) scans and are central to the diagnosis, monitoring, and prediction of osteoporotic fractures. The LS is predominantly composed of trabecular bone and is highly metabolically active, making it sensitive to changes in bone turnover. In contrast, the FN and TH regions contain more cortical bone and are critical predictors of hip fracture risk—a major cause of morbidity, mortality, and healthcare burden in older adults. Evaluating BMD at these regions provides a more precise understanding of how PPIs may influence bone health in clinically meaningful ways.

Some studies have reported outcomes for additional skeletal sites such as the forearm or distal radius; however, these measures are less consistently included in research and are not standard endpoints in osteoporosis diagnosis or treatment monitoring [13, 14]. For this reason, limiting the analysis to the LS, FN, and TH allows for better comparability across studies and enhances the clinical relevance of the findings. By focusing specifically on these three high‐yield DXA sites, this review is aimed at addressing existing knowledge gaps and providing a more definitive synthesis of whether PPI use is associated with measurable reductions in BMD.

This meta‐analysis seeks to provide clarity by systematically evaluating the available evidence on PPI‐associated BMD changes at the LS, FN, and TH. A more precise understanding of these relationships will support evidence‐based clinical decision‐making, guide risk–benefit assessments, and inform recommendations for long‐term PPI management, especially in populations vulnerable to bone loss and fracture, including older adults, individuals with multiple comorbidities, and those already at risk of osteoporosis.

## 2. Material and Methods

### 2.1. Search Strategy

This comprehensive meta‐analysis and systematic literature review article examined the impact of PPI use on LS, FN, and TH BMD. We chose the studies published between January 2010 and January 2025 to examine the change or loss in BMD at these three anatomical locations. In order to acquire pertinent data, the study selection process was conducted in accordance with the guidelines established by the Preferred Reporting Items in Meta‐Analyses (PRISMA) guidelines [21]. The completed PRISMA checklist is provided in Table S3.

**Figure 1:**
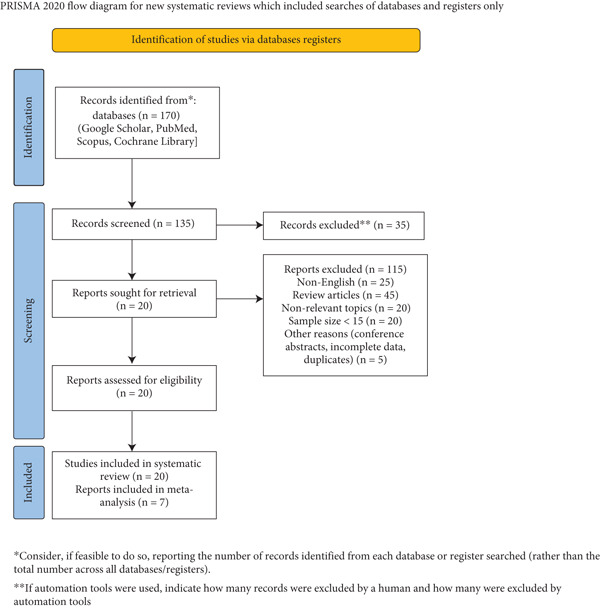
PRISMA 2020 flow diagram.

The systematic review started with 170 records that were identified in databases such as Google Scholar, PubMed, Scopus, and Cochrane Library. Relevant studies were identified by means of specific search terms and filters (Table S1). At the screening phase, 135 records were evaluated, and 35 records were dropped. The 20 remaining reports requested to be retrieved were all retrieved and evaluated to obtain eligibility. Two hundred non‐English articles, 25 review articles, 20 nonrelevant topics, and 20 studies with sample sizes less than 15 were eliminated based on preset criteria. Also, five studies had to be excluded due to other reasons, including being conference abstracts, incomplete data, or duplicates. Finally, 20 studies [22–41] were enrolled in the systematic review. Among these, seven reports were used in meta‐analysis (Figure 1). Data extraction was conducted by two independent reviewers who systematically extracted relevant data from all new studies employing a predesigned data abstraction form. Discrepancies were resolved by discussion and mutual consensus.

### 2.2. Inclusion Criteria

Eligible studies included those examining the effect of PPI use on BMD at the LS, FN, and TH regions. Only peer‐reviewed full‐text publications in the English language were considered. Studies needed to report empirical findings on BMD and include a minimum of 15 patients to ensure robust data.

### 2.3. Exclusion Criteria

Studies were eliminated if they failed to meet the specified criteria. Non‐English publications were excluded to ensure consistency in data interpretation. Review articles, conference abstracts, and studies without empirical findings were also excluded, as they did not provide primary data relevant to the research question. Studies investigating BMD at anatomical locations other than the LS, FN, and TH were excluded. Additionally, research focusing on unrelated conditions, such as the adverse effects of PPIs in individuals with peptic ulcers, Barrett′s esophagus, Zollinger–Ellison syndrome, or those addressing *Helicobacter pylor*i eradication regimens with other comorbidities, was deemed nonrelevant and excluded. Finally, studies with fewer than 15 patients were excluded to ensure the inclusion of robust and reliable data for statistical analysis. This limit was chosen to reduce the risk of bias and improve the reliability of the findings.

### 2.4. Data Extraction

Two evaluators conducted the data extraction process independently based on a standardized and pre‐established data extraction form. The following information was obtained, in relation to each included study:

• Characteristics of the study: author, year of publication, country, and design of the study.
• Participant characteristics: sample size, average age or age range, gender distribution, and pertinent clinical features.
• Intervention specifications: kind of PPI, dose, exposure time, and comparison/control group.
• Interesting outcomes: BMD was assessed in the LS, FN, and TH regions. Where possible, effect sizes, differences in means, and associated measures of variability (standard deviation, standard error (SE), or 95% confidence intervals (CIs)) were extracted.
• Others: length of follow‐up, comorbidity, concomitant medication, and quality/risk of bias measures of the study.

Any inconsistencies identified in the data extracted were addressed through discussion or consultation with a third reviewer. To estimate the effect size, the endpoint data (final BMD values at LS, FN, and TH) were mainly extracted because the data were most consistently reported across studies. Where studies have given change‐from‐baseline values, these were translated to endpoint equivalents or omitted when needed to ensure consistency. When the characteristics of the baselines are balanced (according to Cochrane Handbook rules), endpoint and change data provide a similar outcome.

### 2.5. Management of Missing Data

In cases where there were gaps in the summary statistics in the included studies, the authors were contacted to seek missing information. When no response was obtained, missing standard deviations were estimated based on any available data, CIs, SEs, or *p* values, using standard statistical techniques. In instances where important outcome data (e.g., BMD at a given anatomical location) was unable to be obtained or imputed, the study was not included in the quantitative synthesis but rather subjected to qualitative review. The sensitivity analyses were undertaken to determine the possible effect of missing information on aggregated estimates.

### 2.6. Risk of Bias and Quality Assessment

The assessment of quality and risk of bias methodology, as well as evaluation of bias risk within the incorporated nonrandomized intervention studies, was assessed using the Risk of Bias in Nonrandomized Studies of Interventions (ROBINS‐I) tool [42]. Two reviewers conducted the assessment on their own, and any differences were addressed with a third reviewer.

The ROBINS‐I device measures seven different aspects of bias [42]:

D1: Bias attributed to confounding variables.

D2: Bias arising from participant selection.

D3: Bias associated with the classification of interventions.

D4: Bias resulted from deviations from the intended interventions.

D5: Bias stemming from incomplete data.

D6: Bias in the measurement of outcomes.

D7: Bias in the selection of the reported outcomes.

All of the aforementioned domains were systematically classified according to the level of risk, denoting low, moderate, serious, or critical risk of bias. A study was considered to have a serious overall risk of bias when at least one domain was considered to have a serious risk of bias.

### 2.7. Sensitivity Analysis

**Figure 2:**
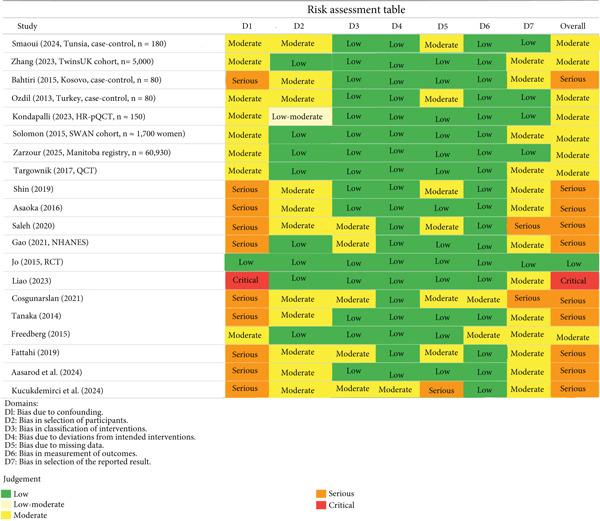
Risk of bias assessment for included studies.

Sensitivity analyses were used to determine the robustness of the aggregated estimates by first sequentially deleting each study (leave‐one‐out analysis) and by only including the studies at low risk of bias in the analysis (Figure 2). Where possible, subgroup analyses were also studied in terms of study design and population characteristics.

### 2.8. Statistical Analysis

R Studio (Version 4.5.1) was used to perform statistical analyses in the meta and metafor packages. Random‐effects models were used to compute the pooled effect sizes to explain between‐study variability. The degree of heterogeneity was assessed with the Cochran *Q* test and quantified through the *I* ^2^ statistic. Funnel plots were employed to visually evaluate the existence of publication bias, while the Egger regression test was used to test the presence of bias statistically. To present the pooled effect sizes and the 95% intervals, forest plots were produced. Sensitivity analyses were conducted to determine the robustness of the aggregated findings. They used a leave‐one‐out method whereby one study was removed at a time to identify how that particular study affects the effect size. Moreover, it became possible to resort to analyses characterized by minimal risk of bias. Subgroup analyses were also contemplated based on study design and population characteristics, where data allowed.

## 3. Results

The present study assesses the impact of PPIs on BMI at three distinct anatomical locations: the LS, FN, and TH regions. In order to achieve validity, studies were included according to the predetermined criteria, including sufficient sample size, the presence of sufficient data to compute the effect sizes, and the quality evaluation with the ROBINS‐I tool. This instrument measures risk of bias across seven domains described in the methodology. To reduce methodological constraints and increase consistency, only studies that had an overall judgment of low, low–moderate, or moderate risk of bias were included in the current risk assessment (Figure 2), whereas studies with a critical risk of bias were excluded.

In the end, seven articles were considered robust enough to be synthesized, and their combined results with respect to PPI use and BMD of the LS, FN, and TH are presented in Table 1.

**Table 1:** Summary of the studies included in the meta‐analysis.

**Theme**	**Smaoui et al. [** **40** **]**	**Zarzour et al. [** **41** **]**	**Bahtiri et al. [** **34** **]**	**Ozdil et al. [** **39** **]**	**Kondapalli et al. [** **37** **]**	**Solomon et al. [** **24** **]**	**Zhang et al. [** **36** **]**
Lumbar spine	Long‐term utilization of PPIs was found to be significantly associated with decreased BMD (*p* value = 0.0031)	No sufficient effect on lumber spine BMD was observed after adjusting for sagittal abdominal diameter	Year‐long use of PPI did not lead to a significant reduction in lumber spine *T*‐scores (*p* value = 0.079)	Lumbar spine BMD *T*‐scores were significantly reduced after at least 6 months of proton pump inhibitor treatment, the *p* value of which was less than 0.01	There were no differences in any of the dual X‐ray absorptiometry parameters, including *T*‐score and trabecular bone score. The result of the lumbar spine *T*‐score was not significant (*p* value = 0.51)	No annualized lumbar spine BMD change difference was found between the PPI users and nonusers	A decrease in the BMD in the lumbar spine was also reported, but this was not statistically significant, and the *p* value was 0.389
Femoral neck	Using PPI shows an insignificant change in femoral neck *T*‐score (*p* value ≤ 0.09)	No sufficient effect on femoral neck BMD was observed after adjusting for sagittal abdominal diameter	The 12 months of PPI treatment showed a significant decrease in the mineral density *T*‐scores of the femoral neck bone (*p* value = 0.005)	The decrease in the BMD *T*‐scores of the femoral neck was reported to be significant after at least 6 months of treatment with PPIs, and the *p* value was 0.01	There was no statistically significant difference in the parameters of dual X‐ray absorptiometry, including the *T*‐score of the femoral neck (*p* value = 0.53)	They found no difference in the change in BMD of the femoral neck in any annualized process between the PPI users and the nonusers	The mineral density of the femoral neck bones was also stated to be significantly reduced, and the *p* value was 0.030
Total hip	Not reported	Not reported	The change in total hip bone mineral density *T*‐scores after 12 months of proton pump inhibitor treatment was significantly reduced (*p* value = 0.001).	Not reported	The parameters of the dual X‐ray absorptiometry, including *T*‐scores of the total hip, did not differ (*p* value = 0.34)	The annualized change in total hip bone mineral density did not differ in proton pump inhibitor users versus nonusers	Mineral density of the total hip bone was found to be significantly reduced (*p* value = 0.00235)

The degree of bias was different in the included studies, with the majority rated as moderate, some as low to low–moderate, and some as serious, mainly because of the inability to adequately control confounding factors, such as age, sex, comorbid conditions, and PPI exposure time. Domains D1 (confounding) and D2 (selection of participants) in particular were often characterized by moderate or serious risks, which are associated with the limitations of the observational design types, as residual confounding due to lifestyle or other factors that are not measurable cannot be disadvantaged. The analysis of the combined results also shows that the results are heterogeneous, which can be attributed to the fact that they were not using the same study design (e.g., case–control/cohort), population demographics (e.g., age, sex, and menopausal status), and exposure duration (typically > 6 months/> 1 year) and that they did not adjust the findings based on the confounding factors (e.g., body mass index/abdominal obesity).

The results of the synthesis of included studies show a dual and sometimes conflicting action of PPI use on BMD. With the LS, two studies identified a significant decline in BMD [39, 40], whereas five studies established no significant difference or reported that the difference was not significant (Table 2) [24, 34, 36, 37, 41]. Likewise, there is inconsistency in the results of the FN with Bahtiri et al. [34], Ozdil et al. [39], and Zhang et al. [36] showing a significant decrease in BMD and Smaoui et al. [40], Kondapalli et al. [37], and Solomon et al. [24] reporting no significant change. The statistics of the TH are restricted as well as contradictory. Significant reduction in BMD was observed by Bahtiri et al. [34] and Zhang et al. [36], but not in Kondapalli et al. [37] and Solomon et al. [24]. In general, part of the evidence points to a relationship between long‐term PPI use and lower BMD, especially of the FN and TH, but the inconsistency of the findings across studies, combined with the absence of a definite dose–response relationship, implies that no single conclusion can be drawn based on the current analysis.

**Table 2:** Literature synthesis.

**Authors**	**Aim**	**Year**	**Methods**	**Findings**	**BMD measurement**	**Country**	**Sample size**	**Age**	**Gender**	**Exposure**	**Control group**	**Treatment duration**
Aasarød et al. [22]	To assess whether the bone quality among patients with GERD exhibits compromised bone quality prior to proton pump inhibitor therapy	2021	Case–control study	The results showed compromised bone quality and inferior BMD in PPI‐naïve GERD patients. Treatment with pantoprazole did not affect bone parameters, indicating that its short‐term use is safe for the skeleton	Dual X‐ray absorptiometry (DXA)	Norway	34 (*C* = 17, intervention = 17)	25–80 years	Male = 12, female = 5	Pantoprazole 40 mg	Age‐ and sex‐matched controls	3 months
Fattahi et al. [23]	To examine an association between prolonged PPI use and BMD	2019	Analytical cross‐sectional study	The study suggests that prolonged usage of PPIs may lead to lower bone mineral density and a higher incidence of osteoporosis in the femoral neck. However, further research with longitudinal evaluation is necessary to establish a causal relationship. It is recommended to avoid excessive use of PPIs due to the potential risk of developing osteoporosis and fractures. Additionally, the use of BMC levels is suggested as a quantitative measure alongside *T*‐scores for analyzing and reporting similar studies	DXA	Iran	394 (PPI users = 133, PPI nonusers = 261)	PPI users = 48.38, PPI nonusers = 47.86	PPI users = 81.2 females, PPI nonusers = 80.5 females	—	—	Median = 6.7 years
Solomon et al. [24]	To explore BMD changes among women initiating PPIs or Histamine 2 receptor antagonists (H2RAs)	2015	Cohort study	No significant difference in annualized BMD change at the lumbar spine, femoral neck, or total hip between PPI users and H2RA users or nonusers	DXA	United States	3302	42–52 years	3302 females	—	—	—
Freedberg et al. [25]	To investigate an association between the use of PPIs with fractures in young adults	2015	Population‐based study	The study found dose–response effect with increased total exposure to PPIs in young adults but not among children, which correlated with the fractures among young adults due to the PPI use. The study concluded that young adults are more prone to develop fractures, although the traditional risk factors for fractures are not present in them. PPIs were identified as a risk factor for developing fractures	PPI–fracture relationship	United Kingdom	730,441 (case = 124,799, control = 605,643)	4–29 years	Cases (male = 81,906, female = 42,893), control (male = 396,129, female = 209,514)	180 cumulative doses of PPIs prior to the index date	5 controls by age, sex, medical practice, and start of follow‐up	—
Tanaka et al. [26]	To find the therapeutic effectiveness of the weekly coadministration of risedronate and PPIs in osteoporosis treatment	2014	Randomized trial	PPI use had no adverse effect on bone metabolism but rather recorded an improvement effect of bodily pain and physical functions	Computer tomography	Japan	96	> 50 years	96 females	17.5 mg dose of sodium risedronate was administered weekly with or without a daily 10 mg dose of sodium rabeprazole	—	24 months
Coşgunarslan et al. [27]	To assess the use of PPIs and the mandibular bone quality	2021	Preliminary retrospective study	In the trabecular and cortical bone, osteoporotic changes were detected in the mental foramen region in PPI users with fractal analysis and morphometric indices. No differences were found for the mandibular ramus and angulus regions	Fractal analysis and panoramic morphometric indices	Turkey	402	Median age values (control = 42, study = 43)	Control (male = 76, female = 125), study (male = 77, female = 124)	—	Patients who were not using any drug which may affect the skeletal system	≥ 1 year
Liao et al. [28]	To find the long‐term use of PPIs and their impact on osteoporotic fractures	2023	Retrospective cohort study	No significant change was observed in BMD among the PPI users	DXA	Taiwan	1518	Mean = 61.3	44.9% Females	Omeprazole, pantoprazole, lansoprazole, and dexlansoprazole	—	≥ 336 days
Jo et al. [29]	To examine the effect of PPIs on bone metabolism mediated by osteoclast action in old age	2014	Prospective randomized study	Bone parameters were assessed in elderly patients. PPIs have the potential to directly alter bone metabolism via the vacuolar H+‐ATPase in osteoclasts	DXA	South Korea	26	55‐85 years	Male = 15, Female = 11	Pantoprazole (40 mg daily) or revaprazan (200 mg daily)	—	8 weeks
Gao et al. [30]	To assess the prolonged use of PPIs on lower BMD in older males	2021	Cross‐sectional study	The differences were found in terms of the association between long‐term use of PPIs and lumbar spine BMD among females and males. Long‐term use of PPIs caused a reduction in BMD of the lumbar spine in older men. No impact of H2RA on lumbar spine BMD was detected	DXA	United States	4232	≥70 years = 42.68, <70 years = 57.32	43.09% males	PPIs and H2RA	—	>1 year = 66.05*%*, ≤1 year = 33.95*%*
Saleh and Al‐Nimer [31]	To figure out changes in the bone mineral density detected by dual‐energy X‐ray absorptiometry in postmenopausal women due to PPIs	2020	Case–control study	Individuals using PPIs exhibit a faster rate of bone loss, regardless of their age, duration of menopause, body mass index, and waist‐to‐height ratio. Based on our analysis, it is concluded that PPI users are more likely to experience bone mass loss in the femur compared to the lumbar vertebrae	DXA	Iraq	215 (control = 150, treatment = 65)	Mean = 60.3	215 women	Patients treated with PPIs (omeprazole, lansoprazole, and esomeprazole)	No treatment with PPIs	—
Asaoka et al. [32]	To find the efficacy of alfacalcidol (AC) and alendronate (AD) on lumbar bone mineral density in osteoporotic patients using PPIs	2016	Prospective, randomized, active control study	The study showed that osteoporotic patients who took both PPIs and AD had a more significant increase in lumbar BMD after 1 year of treatment compared to those who took AC. However, the study had a limited number of participants, so large prospective studies are necessary to confirm the effect of AD in osteoporotic patients taking PPIs	DXA	Japan	16	Mean = 64.1 years	Male = 4, female = 12	Alfacalcidol (1 *μ*g/day), alendronate (35 mg/week) in PPI users	—	1 year
Shin et al. [33]	To assess the link between lower trabecular bone score (TBS) and the use of PPIs	2019	Study from medical records	The use of PPIs may affect trabecular bone quality early but may be reversible, as suggested by the association of lower TBS with current PPI use	DXA	South Korea	1505	40–89 years	Female = 1505	Subjects who had received PPI prescriptions	No PPI prescriptions	—
Bahtiri et al. [34]	To examine the use of esomeprazole in the reduction of BMD	2016	1‐year prospective comparative safety study	After 12 months of PPI treatment, the femoral neck and total hip BMD *T*‐scores were lower compared to the baseline. Among the four PPIs examined, esomeprazole was found to have a significant impact on BMD reduction, while omeprazole had no effect on BMD. Given the widespread use of PPIs, it is recommended to consider BMD screening for people who use PPIs for an extended period	DXA	Republic of Kosovo	209 (exposure = 167, control = 42)	18–65 years	156 females	Oral PPIs	No use of PPIs	—
Targownik et al. [35]	To find long‐term PPI use and its potential link with changes in bone strength and structure	2017	Cohort study	There is no evidence to suggest that long‐term use of PPIs is linked to any changes in BMD or strength that may increase the risk of fractures. This indicates that the connection between PPI use and fractures is not a direct cause	DXA	Canada	102	Mean = 65.1	PPI users = 54 females, PPI nonusers = 48 females	PPI usage	No PPI usage	> 180 days/year in each of the 5 years
Zhang et al. [36]	To investigate whether PPI‐induced changes in plasma metabolite levels influence total hip bone mineral density in a UK cohort	2020	UK cohort study	The use of PPIs may have an impact on total hip BMD, both directly and indirectly, via plasma metabolites involved in the sex hormone pathway	DXA	United Kingdom	7738 (PPI users = 1292, PPI nonusers = 6446)	Mean = 51.88	Male = 692, female = 4972	PPI usage	No PPI usage	—
Kondapalli et al. [37]	To measure the microstructure and stiffness of the skeletal structure	2023	Cross‐sectional study	No difference in bone microstructure or bone stiffness was observed with the use of proton pump inhibitors (PPIs), but women using PPIs had more falls	DXA (LS, FN, TH, radius), TBS, VFA, HRpQCT, *μ*FEA	United States	601 (130 users, 471 nonusers)	> 65 years (mean ~76.7)	402 women, 199 men	Daily/intermittent PPIs	Nonusers	Not specified
Kucukdemirci et al. [38]	To determine the bone changes in young women on short‐term PPIs	2024	Retrospective cohort study	Six to 12 months of PPI use led to the reduction of *T*‐scores and *Z*‐scores, but this was not enough to achieve the osteopenia threshold; PPI use for less than 6 months showed no real effect	DXA (FN *T*/*Z*‐scores)	Turkey	117 females	20–40 years (mean 32.8 ± 5.3)	Female	PPIs < 6 or 6–12 months	Nonusers	< 6 or 6–12 months
Ozdil et al. [39]	To examine the effect of PPIs on bone density	2013	Prospective study	PPIs also had links to a decrease in lumbar spine and femoral neck bone mineral density *T*‐scores, with the strongest effect being on esomeprazole	DXA (LS, FN)	Turkey	224 (114 PPIs, 110 controls)	18–56 years (mean 37.7 ± 8.8)	51.8% female	Lansoprazole, esomeprazole, pantoprazole ≥ 6 months	Healthy controls	≥ 6 months
Smaoui et al. [40]	To determine the effects of long‐term PPIs on phosphocalcium metabolism and BMD	2024	Case–control study	A reduction in bone mineral density was also associated with long‐term use of PPIs, with the risk of this effect being increased in people older than 50 years old, postmenopausal women, and those who take more than two doses of the medication	Osteodensitometry (spine, femur *T*‐scores)	Tunisia	180 (90 PPIs, 90 controls)	18–80 years (mean 55 ± 12.2)	50 females (55.6%)	PPI > 1 year	Nonusers	> 1 year
Zarzour et al. [41]	To determine the PPI exposure, TBS, and BMD with SAD adjustment	2025	Registry‐based cohort study, Manitoba BMD Registry	There was a positive correlation between PPI use and sagittal abdominal diameter, though there was no significant association between the use of PPI and trabecular bone score or bone mineral density when confounding variables were controlled	DXA (LS, FN), TBS	Canada	60,930 (11,340 PPI; 49,590 nonusers)	Mean 65.7 years	90.3% women	PPI use categorized by MPR	Nonusers	12‐month baseline, 3.4‐year follow‐up

The meta‐analysis, comprising seven distinct studies [24, 34, 36, 37, 39–41], revealed that the negative pooled effect was statistically significant across all the anatomical sites as the standardized mean difference (SMD) was −0.15 (95% CI: [−0.21, −0.09]). This overall finding was moderated by the existence of such high and statistically significant heterogeneity (*I* ^2^ value of 93.6%). To examine this, a subgroup analysis was conducted and found that the site of the study was a key factor in the heterogeneity. The pooled effect of −0.27 (95% CI: [−0.46, −0.09]) of the FN subgroup was statistically significant, and the pooled effects of the LS and TH subgroups were not statistically significant. This means that although there is a negative pooled effect across the studies, this effect is not uniform, and it is specifically strong in the FN.

**Figure 3:**
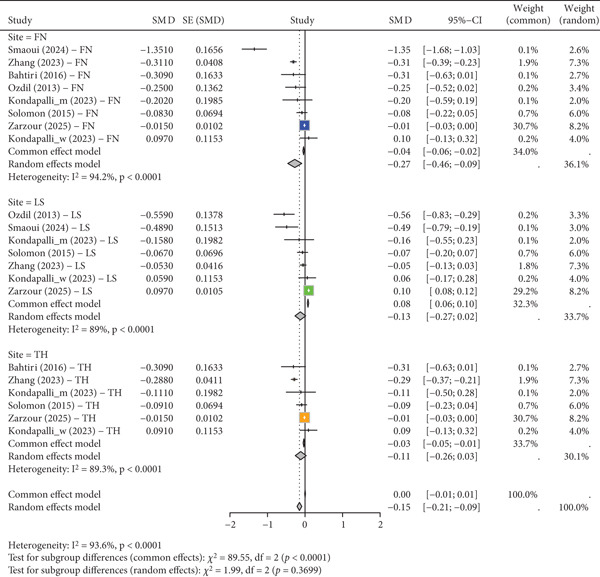
Forest plot illustrating the standardized mean difference (SMD) and 95% confidence intervals (CIs) of PPI use on bone mineral density (BMD). The data is separated into three subgroups: femoral neck (FN), lumbar spine (LS), and total hip (TH). The heterogeneity (*I* ^2^) is presented for each subgroup and for the overall effect. The diamond at the bottom of each section represents the pooled effect size, calculated using both common‐effects and random‐effects models. The weight of each study in the analysis is also displayed. The overall pooled SMD is shown at the bottom.

The meta‐analysis asymmetry and heterogeneity sources are further explained by a finer funnel plot with study labels as illustrated in Figure 3. It can be seen that there is an asymmetry in the plot (pictured above), which shows the SMD on the *x*‐axis versus the SE on the *y*‐axis. Most of the studies are concentrated on the left side, specifically those that have smaller effect sizes and less precision, which could represent a reporting bias toward negative results. In particular, the plot shows that Smaoui et al. [40] for FN stands out as a definite exception, being located significantly to the left of the rest of the studies. This supports our earlier discussion that this study alone is a significant source of the high heterogeneity and asymmetry. Also, research having very high accuracy, as Zarzour et al. [41] for FN and Zarzour et al. [41] for LS, is very near the center line of no effect, indicating the possibility of variation in study factors that could affect the outcome. This visualization goes a long way to confirm the conclusion that the overall pooled effect must be taken with caution and that the observed heterogeneity cannot be attributed to randomness.

**Figure 4:**
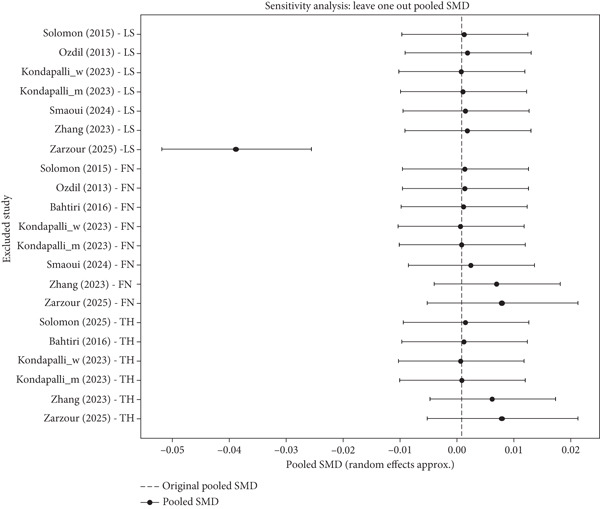
Sensitivity analysis (leave‐one‐out analysis) illustrating pooled standardized mean difference.

**Figure 5:**
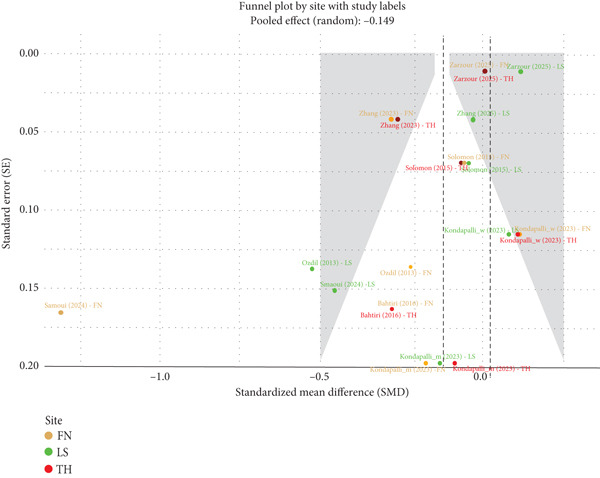
Funnel plot illustrating potential publication bias by site. The plot is separated by study site: femoral neck (FN), lumbar spine (LS), and total hip (TH).

To determine the strength of the accumulated effect size, the sensitivity analysis, leave‐one‐out pooled SMD, was done sequentially, leaving out one study at a time and recalculating the pooled SMD (Figure 4). The aim here was to find out whether one study is heavily affecting the overall outcome. The initial pooled SMD is 0.0010 as shown in Figure 5 and Table S2 with a 95% CI, which is −0.0101 to 0.0120. This CI is not 0, which means no statistically significant global effect. It can be seen that the results of the overall analysis are very robust and not dependent on any individual study, with one exception. In both cases where either study is excluded, the new pooled SMD and 95% CI tend to be extremely similar to the initial pooled SMD and to cross the zero line, showing that there is no significant effect. The interesting exception is the Zarzour et al. [41] for LS. Removal of this study alters the pooled SMD at a significant level of −0.0386 and 95% CI of −0.0518 to −0.0255. This new CI does not pass through 0, indicating that elimination of this particular study results in a statistically significant negative pooled effect. This observation implies that the Zarzour et al. [41] for the LS study is an outlier and has a large impact on the aggregate pooled effect, concealing a potentially large negative effect that is only evident when this outlier is removed. This justifies a more in‐depth review of the Zarzour et al. [41] study to better comprehend why its results vary so significantly compared to the rest and why it seems to be such a significant source of the overall null result. The heterogeneity measures (*Q*, *I* ^2^, and Tau^2^) are identical to those of all of the leave‐one‐out analyses, which means that dropping a single study does not materially alter the high degree of heterogeneity in the meta‐analysis.

## 4. Discussion

Generally, an enhanced risk of fracture is correlated with the administration of PPIs among patients with GERD. Aasarød et al. [22] investigated the BMD at the LS, FN, TH, and trabecular bone score (TBS) in order to evaluate the utilization of PPIs as a potential risk factor for bone impairment. It was a case–control study in which 17 participants were in each group. The BMD was measured utilizing DXA. Based on the findings, BMD was found to be lower at the TH and FN in patients of the case group in comparison with the control group. However, this BMD reduction was not statistically significant at the TH and FN (*p* = 0.09 and 0.12, respectively). To conclude, a significant reduction in PPI use was found, where, for the short term, its usage was not reported to cause compromised bone quality. Fattahi et al. [23] assessed the chronic use of PPIs as a risk factor for developing osteoporosis and reduction or alterations in the BMD. This cross‐sectional study recruited patients who had been using PPIs for the last 2 years. Based on DXA, a highly significant reduction was reported on the FN (*p* < 0.001). The study concluded that patients who had been using PPIs for years had a high likelihood of developing a reduction in BMD in the FN and osteoporosis. However, no significant differences were documented among the patients of the case and control groups in terms of reduction in the BMD of the TH and LS.

The overuse of PPIs is a potential cause of developing osteoporosis and a reduction in BMD. In the study of Liao et al. [28], no significant differences were found among CYP2C19 genotypes and fracture, and the use of PPIs was not correlated with any reduction in the BMD of the LS, FN, and TH. Shin et al. [33] studied the effects of PPIs on the TBS. In this cohort study, the medical records of 1505 women who were aged 40–89 years were examined to assess their BMD. Among them, a total of 233 women were given PPIs. The findings revealed that in the PPI exposure group, there was an apparent reduction in the BMD levels of the FN, LS, and TH among those women. These results showed that bone quality is potentially reduced by PPI use, however with the reversible effects. Bahtiri et al. [34] demonstrated that a duration of 12 months of PPI administration was correlated with a statistically significant decrease in FN (*Z* = −2.764, *p* = 0.005), TH (*Z* = −3.281, *p* = 0.001), and LS (*p* = 0.048) measurements.

Among the four investigations concerning PPIs, esomeprazole was found independently related to a substantial reduction of BMD; thus, the study advocated for BMD screening across the wide use of PPIs during prolonged administration. The findings of Targownik et al. [35] examined the correlation between PPI utilization and the incidence of fractures through the application of three‐dimensional quantitative computed tomography (3D‐QCT) imaging techniques. This cohort study compared the patient (*n* = 52) data of long‐term PPI use (≥ 5 years) with those with no history of using PPIs (*n* = 52). The results of the study validated no discernible impact of long‐term PPI use based on standard BMD, volumetric BMD, markers of bone metabolism, or measures of bone strength of FN, LS, and TH, as there was no significant difference between the BMD levels of patients in both groups. Furthermore, these findings suggested the absence of a causal relationship between the utilization of PPIs and the incidence of fracture. A significant strength of this meta‐analysis is that it directly compared the effect of the use of PPIs on the BMD at the LS, FN, and TH, three anatomical locations that were not reviewed comprehensively in the earlier meta‐analyses. With these clinically relevant areas in focus, our study contributes to the knowledge on the site‐specific PPIs on bone health and offers more comprehensive evidence to help clinicians evaluate the skeletal risk of PPI users.

Our study evaluates the effect of PPI use on BMD across three anatomical sites that were not included in previously published meta‐analyses. Nevertheless, there are certain limitations for consideration. Firstly, only three anatomical sites were selected for the meta‐analysis, which may constrain the generalizability of our results. Furthermore, publication bias was assessed using only seven studies, whereas a greater number of studies is deemed to yield greater reliability. Additionally, the search strategy could be enhanced by including specific names of PPIs to ensure comprehensive coverage. Ultimately, conducting a search exclusively focused on “BMD” without inclusion of related terms such as “fracture,” “osteoporosis,” or other bone health–related keywords may have excluded studies where BMD was not the primary outcome but was reported within the main body of text. Future studies could include a broader range of anatomical sites, expand the search strategy, and incorporate a larger number of studies to more accurately assess publication bias and further validate these findings.

## 5. Conclusion

The present study concludes that despite reported adverse impacts of the PPI use, there was an absence of statistically significant impact of the PPI use on the BMD level at the specified anatomical locations, as no study included in the analysis recorded a statistically significant reduction in the mineral density of these bony structures. Subsequent research endeavors may further explore differences in PPI effects based on age, sex, menopausal status, or underlying conditions to elucidate how these factors influence BMD across different demographics and clinical characteristics.

## Consent

The authors have nothing to report.

## Disclosure

All authors approved the final manuscript as submitted and accepted responsibility for all aspects of the work.

## Conflicts of Interest

The authors declare no conflicts of interest.

## Author Contributions

A.A.A. and B.A.B. contributed to the composition and design of the study; carried out the systematic literature search, quality assessment, and meta‐analysis and interpretation; drafted the initial manuscript; and reviewed and amended it for final approval. F.M.A. contributed to data collection and initial manuscript drafting.

## Funding

No funding was received for this manuscript.

## Supporting Information

Additional supporting information can be found online in the Supporting Information section.

## Supporting information


**Supporting Information 1** Table S1: Search strategy.

**Supporting Information 2** Table S2: Sensitivity analysis (leave‐one‐out) of included studies.

**Supporting Information 3** Table S3: PRISMA 2020 checklist.

## Data Availability

All the information in this study is available in the full‐text articles of this systematic review and meta‐analysis. The basic study data is accessible upon request to the corresponding author.
