# Supplementary material for: The Effect of Proton Pump Inhibitors on Bone Mineral Density at Specific Anatomical Sites: A Systematic Review and Meta‐Analysis
Source: Biomed Res Int. 2025 Dec 29;2025:1269905. doi: 10.1155/bmri/1269905 (PMC12748532; doi:10.1155/bmri/1269905)
Supplement: Supplementary file 1 — Supporting Information 1 Table S1: Search strategy. [file BMRI-2025-1269905-s002.docx]

**Supplementary Material**

**Table S1. Search Strategy**

| **Database** | **Search Terms** | **Filters** |
| --- | --- | --- |
| **PubMed** | #1 Proton Pump Inhibitors: ("Proton Pump Inhibitors"[Mesh] OR "PPI"[tiab] OR "proton pump inhibitor"[tiab])  #2 Bone Mineral Density: ("Bone Density"[Mesh] OR "BMD"[tiab] OR "bone mineral density"[tiab])  #3 Anatomical Sites: ("Lumbar Spine"[tiab] OR "Femoral Neck"[tiab] OR "Total Hip"[tiab])  #4 Combination: #1 AND #2 AND #3 | Language: English  Publication Year: **2010–2025**  Exclude animal studies: NOT ("Animals"[Mesh] NOT "Humans"[Mesh]) |
| **Scopus** | Proton Pump Inhibitors: TITLE-ABS-KEY("proton pump inhibitors" OR "PPI")  Bone Mineral Density: TITLE-ABS-KEY("bone density" OR "BMD" OR "bone mineral density")  Anatomical Sites: TITLE-ABS-KEY("lumbar spine" OR "femoral neck" OR "total hip") | Document Type: Article  Language: English  Publication Year: **2010–2025** |
| **Cochrane Library** | Proton Pump Inhibitors: ("proton pump inhibitors" OR "PPI")  Bone Mineral Density: ("bone density" OR "BMD" OR "bone mineral density")  Anatomical Sites: ("lumbar spine" OR "femoral neck" OR "total hip")  Combination: #1 AND #2 AND #3 | Study Type: Randomized Controlled Trials (RCTs)  Language: English  Publication Year: **2010–2025** |
| **Google Scholar** | ("proton pump inhibitors" OR "PPI") AND ("bone density" OR "BMD") AND ("lumbar spine" OR "femoral neck" OR "total hip") | Sorted by relevance.  Reviewed the first 200 results for eligibility.  Publication Year: **2010–2025** |
