# Supplementary material for: The Effect of Proton Pump Inhibitors on Bone Mineral Density at Specific Anatomical Sites: A Systematic Review and Meta‐Analysis
Source: Biomed Res Int. 2025 Dec 29;2025:1269905. doi: 10.1155/bmri/1269905 (PMC12748532; doi:10.1155/bmri/1269905)
Supplement: Supplementary file 2 — Supporting Information 2 Table S2: Sensitivity analysis (leave‐one‐out) of included studies. [file BMRI-2025-1269905-s001.zip › BioMed RI-Supplement Table 2-Sensit_2.docx]

**Table S2.** Sensitivity Analysis (Leave-One-Out) of Included Studies

| **Excluded Study** | **Pooled SMD** | **95% CI (Lower–Upper)** | **Q** | **df** | **p-value** | **I² (%)** | **Tau²** |
| --- | --- | --- | --- | --- | --- | --- | --- |
| Solomon (2015) - LS | 0.0014 | -0.0097 to 0.0125 | 310.84 | 20 | <0.001 | 93.6 | 0.0128 |
| Ozdil (2013) - LS | 0.0019 | -0.0092 to 0.0130 | 310.84 | 20 | <0.001 | 93.6 | 0.0128 |
| Kondapalli_w (2023) - LS | 0.0008 | -0.0103 to 0.0119 | 310.84 | 20 | <0.001 | 93.6 | 0.0128 |
| Kondapalli_m (2023) - LS | 0.0011 | -0.0100 to 0.0122 | 310.84 | 20 | <0.001 | 93.6 | 0.0128 |
| Smaoui (2024) - LS | 0.0017 | -0.0094 to 0.0127 | 310.84 | 20 | <0.001 | 93.6 | 0.0128 |
| Zhang (2023) - LS | 0.0020 | -0.0092 to 0.0132 | 310.84 | 20 | <0.001 | 93.6 | 0.0128 |
| Zarzour (2025) - LS | -0.0386 | -0.0518 to -0.0255 | 310.84 | 20 | <0.001 | 93.6 | 0.0128 |
| Solomon (2015) - FN | 0.0015 | -0.0096 to 0.0126 | 310.84 | 20 | <0.001 | 93.6 | 0.0128 |
| Ozdil (2013) - FN | 0.0014 | -0.0097 to 0.0125 | 310.84 | 20 | <0.001 | 93.6 | 0.0128 |
| Bahtiri (2016) - FN | 0.0013 | -0.0097 to 0.0124 | 310.84 | 20 | <0.001 | 93.6 | 0.0128 |
| Kondapalli_w (2023) - FN | 0.0007 | -0.0104 to 0.0118 | 310.84 | 20 | <0.001 | 93.6 | 0.0128 |
| Kondapalli_m (2023) - FN | 0.0011 | -0.0099 to 0.0122 | 310.84 | 20 | <0.001 | 93.6 | 0.0128 |
| Smaoui (2024) - FN | 0.0025 | -0.0085 to 0.0136 | 310.84 | 20 | <0.001 | 93.6 | 0.0128 |
| Zhang (2023) - FN | 0.0071 | -0.0041 to 0.0183 | 310.84 | 20 | <0.001 | 93.6 | 0.0128 |
| Zarzour (2025) - FN | 0.0080 | -0.0053 to 0.0213 | 310.84 | 20 | <0.001 | 93.6 | 0.0128 |
| Solomon (2015) - TH | 0.0016 | -0.0095 to 0.0127 | 310.84 | 20 | <0.001 | 93.6 | 0.0128 |
| Bahtiri (2016) - TH | 0.0013 | -0.0097 to 0.0124 | 310.84 | 20 | <0.001 | 93.6 | 0.0128 |
| Kondapalli_w (2023) - TH | 0.0008 | -0.0103 to 0.0118 | 310.84 | 20 | <0.001 | 93.6 | 0.0128 |
| Kondapalli_m (2023) - TH | 0.0011 | -0.0100 to 0.0121 | 310.84 | 20 | <0.001 | 93.6 | 0.0128 |
| Zhang (2023) - TH | 0.0065 | -0.0046 to 0.0177 | 310.84 | 20 | <0.001 | 93.6 | 0.0128 |
| Zarzour (2025) - TH | 0.0080 | -0.0053 to 0.0213 | 310.84 | 20 | <0.001 | 93.6 | 0.0128 |
| **Overall (no exclusion)** | **0.0010** | **-0.0101 to 0.0120** | **310.84** | **20** | **<0.001** | **93.6** | **0.0128** |
